# Mass Transfer from Ion-Sensing Component-Loaded Nanoemulsions into Ion-Selective Membranes: An Electrochemical Quartz Crystal Microbalance and Thin-Film Coulometry Study

**DOI:** 10.1021/acsmeasuresciau.2c00053

**Published:** 2022-10-04

**Authors:** Canwei Mao, Yoshiki Soda, Kye J. Robinson, Tara Forrest, Eric Bakker

**Affiliations:** Department of Inorganic and Analytical Chemistry, University of Geneva, Quai Ernest-Ansermet 30, CH-1211Geneva, Switzerland

**Keywords:** nanoemulsion, thin-film voltammetry, ion-selective membrane, doping of sensing components, electrochemical quartz crystal microbalance

## Abstract

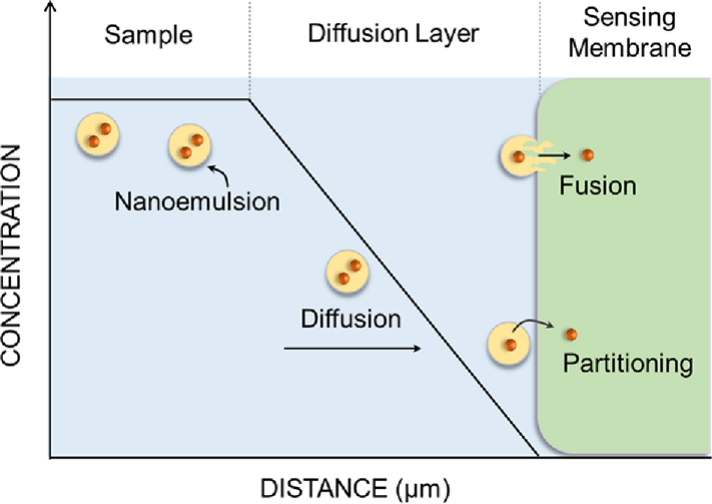

Recent work has shown that ion-selective components may be transferred from nanoemulsions (NEs) to endow polymeric membranes with ion-selective sensing properties. This approach has also been used for nanopipette electrodes to achieve single-entity electrochemistry, thereby sensing the ion-selective response of single adhered nanospheres. To this date, however, the mechanism and rate of component transfer remain unclear. We study here the transfer of lipophilic ionic compounds from nanoemulsions into thin plasticized poly(vinyl chloride) (PVC-DOS) films by chronoamperometry and quartz crystal microbalance. Thin-film cyclic coulovoltammetry measurements serve to quantify the uptake of lipophilic species into blank PVC**-**DOS membranes. Electrochemical quartz crystal microbalance data indicate that the transfer of the emulsion components is insignificant, ruling out simple coalescence with the membrane film. Ionophores and ion-exchangers are shown to transfer into the membrane at rates that correlate with their lipophilicity if mass transport is not rate-limiting, which is the case with more lipophilic compounds (calcium and sodium ionophores). On the other hand, with less lipophilic compounds (valinomycin and cation-exchanger salts), transfer rates are limited by mass transport. This is confirmed with rotating disk electrode experiments in which a linear relationship between the diffusion layer thickness and current is observed. The data suggests that once the nanoemulsion container approaches the membrane surface, transfer of components occur by a three-phase partition mechanism where the aqueous phase serves as a kinetic barrier. The results help better understand and quantify the interaction between nanoemulsions and ion-selective membranes and predict membrane doping rates for a range of components.

Nanoemulsions (NEs) are applied extensively in diverse domains such as drug delivery, food production, and fundamental research.^1−4^ These applications typically rely on the partitioning of components of interest between the organic and aqueous phase. This can be explained by the fact that these nanoemulsions can separate two phases exhibiting different polarities. As an example, some ion-selective optical nanosensors are based on a spectral absorption shift of a solvatochromic dye induced by the solution polarity difference that is driven by the concentration change of the ion of interest.^5,6^ Single-entity electrochemistry also utilizes encapsulated NEs in combination with a redox probe to study single-particle collision events during electron or ion transfer reactions.^4,7^

Recently, our group proposed a new approach to prepare ion-selective membranes (ISMs) that relies on membrane doping through nanoemulsions loaded with appropriate sensing components.^8^ This principle was suggested after observing the contamination of such membranes by lipophilic ionic species contained in microemulsions.^9^ To date, however, the mass transfer mechanism and associated rate-limiting step of transferring components from the nanoemulsion to the membrane is still not understood.

This work evaluates two hypotheses on how components from the nanoemulsion phase may be transferred to the polymeric membrane phase, which are illustrated in Scheme 1. They include two steps that may each become rate-limiting, namely, (a) mass transport from the solution bulk to the membrane surface as the first step followed by either (b) the direct fusion of the nanoemulsion body and the membrane or (c) transfer by partitioning via the intermediate aqueous phase in the microgap between the nanoemulsion and the membrane. Nanoemulsions form a metastable system owing to steric hindrance and overlapping diffuse layers that restrict interparticle coalescence.^10^ These forces should inhibit the direct fusion of nanoemulsions with the membrane, although compositional ripening^11^ may be similar to the direct fusion shown in (b). In mechanism (c), the transfer across three phases (NE–water–membrane) is considered where the interaction with the aqueous phase will modulate the transfer rate.^9^

**Scheme 1:**
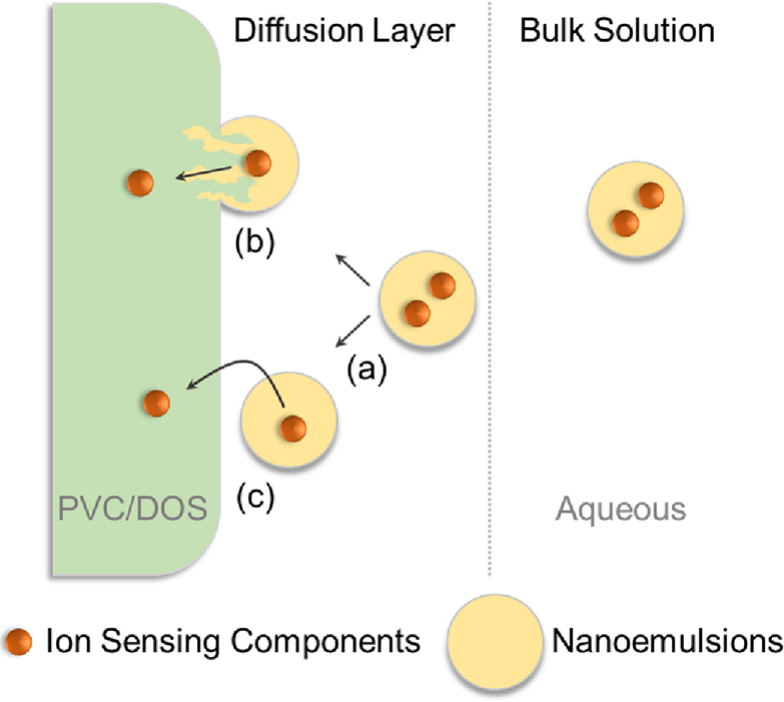
Hypothesis of the Transfer Mechanism of Nanoemulsions Delivering Their Cargo to an Ion-Selective Membrane The rate-limiting step is postulated to be either (a) diffusional mass transport of the nanoemulsion, (b) fusion of the entire nanoemulsion phase with the membrane, or (c) delivery of the nanoemulsion components into the membrane by a partitioning mechanism.

Methods to study the transfer mechanism of emulsions include HPLC,^12^ optical measurements,^11^ and electrochemistry,^13^ which rarely focus on studying the mass transfer of molecules across three phases in real time. This work describes our efforts to introduce a new technique to achieve this. Thin-film cyclic coulovoltammetry (TFCV) for observing multiple species transfer is proposed here in contrast to potentiometry^8^ or chronoamperometry with thick membranes.^14^ To achieve this, a sub-micrometer thin polymeric film deposited onto a solid ion transducing substrate was chosen as the receiving membrane phase, which is in contrast to our previous work that used a classical ion-selective membrane of ca. 200 μm thickness.^8^ The finite thickness of the membrane allows for an exhaustive turnover of the available ion-exchanger sites in the membrane while sweeping the potential. This, together with the peak potential of the ion transfer process gives direct information on the mass transfer rate of a range of active components from the emulsion phase.^15^ Other conventional methods cannot provide this information as easily.

Additional methods used here to better understand the transfer mechanism include electrochemical quartz crystal microbalance (EQCM) as it allows one to obtain mass changes of the overlaying membrane layer^16,17^ and study the mass transfer process of both electrochemically inactive and active species.^18^ EQCM data further help us to understand the transfer mechanism hypotheses shown in Scheme 1.

This work uses nanoemulsions prepared with *N*,*N*-dimethylformamide (DMF) as solvent due to its ability to transfer a number of structurally different ionophores as shown in previous work.^8^ The diffusion layer thickness is controlled by a rotating disk electrode to better understand mass transport kinetics of the emulsion solution.^19^ This allows one to provide an adequate model for emulsion transfer to better understand and predict the uptake rate of ion-sensing components.

## Experimental Section

### Chemicals and Instruments

3-[(3-Cholamidopropyl)dimethylammonio]-1-propanesulfonate (CHAPS), tetrabutylammonium chloride (TBACl), tetramethylammonium chloride (TMACl), potassium chloride (KCl), sodium chloride (NaCl), lithium chloride (LiCl), calcium chloride (CaCl_2_), sodium hexafluorophosphate (NaPF_6_), bis(2-ethylhexyl) sebacate (DOS), poly(vinyl chloride) (PVC), sodium tetrakis[3,5-bis-(trifluoromethyl)phenyl]borate (NaTFPB), tetrahydrofuran (Selectophore, THF), *N*,*N*-dimethylformamide (DMF), valinomycin (K-I), 4-*tert*-butylcalix[4]arene-tetraacetic acid tetraethyl ester (Na-X), and *N*,*N*-dicyclohexyl-*N*′,*N*′-dioctadecyl diglycolic diamide (Ca-IV) were purchased from Sigma-Aldrich. Solutions were prepared with deionized (DI) water (∼18.2 MΩ·cm). Acetonitrile (ACN) was bought from Fischer Scientific. 2-*n*-tetradecyl-2,3-dihydrothieno-[3,4-*b*][1,4]dioxine (EDOT-C_14_) was synthesized in house as previously reported.^20^

Glassy carbon (GC) electrodes (diameter ca. 3 mm) and AT-cut quartz crystal gold-coated electrodes (diameter ca. 7 mm) were purchased from Metrohm (Switzerland). Spin coating of membranes on GC electrodes was performed using a LabSpin instrument from SUSS MicroTec. Limiting the diffuse layer thickness was achieved using a rotating disk electrode (RDE, Metrohm Autolab B.V., Utrecht, Netherlands). Electrochemical experiments were performed with a PGSTAT 101, and the electrochemical quartz crystal microbalance (EQCM) was controlled by the 6 MHz crystal oscillator (Metrohm Autolab B.V., Utrecht, Netherlands), both governed by Nova 2.1 software. This experiment involved a double-junction Ag/AgCl/3 M KCl/1 M LiOAc reference electrode and a platinum electrode, except for EQCM, which used a gold coil counter electrode and an Ag/AgCl (3 M KCl) reference electrode (all electrodes mentioned here were bought from Metrohm, Switzerland). Wolfram Mathematica 12 software was used for theoretical calculations and data treatment.

### Electrode Preparation

PEDOT-C_14_ (10 mM EDOT-C_14_, 30 mM NaPF_6_ in ACN) was first electro-deposited onto polished GC electrodes by cyclic voltammetry over two cycles between −0.8 and 1.35 V at a scan rate of 0.1 V s^–1^ (for the quartz crystal gold electrode, three cycles of cyclic voltammetry were applied). Following the deposition, the electrode was kept in pure ACN for 30 min to remove unbound electrolyte and monomer residues. Afterward, 25 μL of membrane cocktail solution (33% *w*/*w* PVC and 66% *w*/*w* DOS of total mass in THF with a dilution factor of 4) was spin-coated onto the electrode surface at 1500 rpm for 2 min to obtain the thin ISM electrode.

### Mechanistic Ion Transfer Study

Nanoemulsions: Nanoemulsions were generally prepared according to the procedure described in reference (8). DMF-based nanoemulsions were prepared by injecting DMF containing the appropriate sensing components into aqueous electrolyte solution at stirring rate of about 300 rpm, except for EQCM experiments where the reaction cells could not be stirred. Component concentrations and volume ratios of solvent to solution are given below, but generally 20 μL DMF solution was injected into a volume of 2.00 mL. For CHAPS-based nanoemulsions prepared for EQCM experiments, 0.5 mg of PVC, 1 mg of DOS and ion-sensing components were first dissolved in 100 μL of THF, then added to 1 mL of electrolyte solution containing surfactant (CHAPS, 0.1 mg/mL) while stirring at 300 rpm. After evaporating THF for 2 h with air streaming above this emulsion solution, it was injected into 1 mL of solution with electrolytes to the EQCM reaction cell (for specific concentrations see below).

EQCM: The AT-cut quartz crystal gold/PEDOT-C_14_/membrane-coated electrode was fixed between two O-rings (FFKM, KALREZ, high chemical resistance to THF and DMF) in the EQCM cell tightened by three screws. The top of the cell had three holes for connecting the reference and counter electrodes and a spare one for sample injection. After 15 min of the EQCM warming up in 2 mL of DI water/solution containing 10 mM LiCl, the PVC/DOS membrane took ca. 2 h to stabilize. Then, in order to confirm the stability of nanoemulsions, 20 μL of DMF was added in 2 mL of DI water or 1 mL of CHAPS-based emulsion solution (0.5 mg of PVC, 1 mg of DOS, and 100 μL of THF mixed in ca. 1 mL of 0.1 mg mL^–1^ CHAPS solution then evaporated THF under air stream for 2 h) in 1 mL of DI water when only recording the frequency. Additionally, the chronoamperometry measurements were performed at 0.4 V with the same protocol as above but initiated with a solution containing 10 mM LiCl instead of DI water. The emulsion solution (0.2 mg NaTFPB + 20 μL of DMF or 0.5 mg of NaTFPB + 0.5 mg of PVC + 1 mg of DOS + 1 mL of 0.1 mg mL^–1^ CHAPS) was then injected into the cell solution (2 mL or 1 mL) followed by 20 μL of solution with 1 M NaCl, TMACl, and TBACl sequentially every 10 min.

Chronoamperometry with RDE: The GC/PEDOT-C_14_ electrode coated with only the PVC/DOS membrane was immersed in 10 mL of DMF-based emulsion (0.5 mg of NaTFPB, 100 μL of DMF, 10 mM TBACl) while applying a constant voltage of 0.4 V. After a 200 s stabilization period in stagnant solution, the rotating speed of the electrode was increased to 100, 200, and 300 rpm and then decreased to 200, 100, and 0 rpm with an interval time of 50 s.

Cyclic voltammetry(CV) with RDE: The GC/PEDOT-C_14_ electrode coated with only the PVC/DOS membrane in 10 mL of DMF-based emulsion (0.25 mg of NaTFPB, 50 μL of DMF, 10 mM TBACl) was investigated by CV from −0.6 to 0.4 V at a 0.2 V s^–1^ scan rate for two cycles, after which the potential was held at −0.6 V. Every 2 min, the same protocol was repeated for a total time of 20 min. Each set of experiments used three replicates at each rotating speed measured separately, namely, 100, 200, 300, 400, and 500 rpm. The PVC/DOS membrane doped with different components followed the same protocol as above, but the rotating speed was kept at 100 rpm for all cases. The composition of the emulsion with only ionophores (K-I, Na-X, or Ca-IV) was 0.25 mg of ionophores in 50 μL of DMF mixed with 10 mL of solution containing 10 mM of the corresponding ion for which the ionophore is selective (K^+^, Na^+^, or Ca^2+^).

Dynamic light scattering (DLS): The size of NEs was measured by DLS at a 173° scattering angle with a zetaSizer Nano ZS apparatus (Malvern Instruments). Based on the Stokes–Einstein relationship, the size was calculated by the scattered light intensity for three different replicates.

The calculated partitioning coefficients (log *P*) were calculated by ChemDraw 21.0.0, and the volume of each molecule (*V*_COS_) was estimated by a COSMO calculator.

## Results and Discussion

**Scheme 2:**
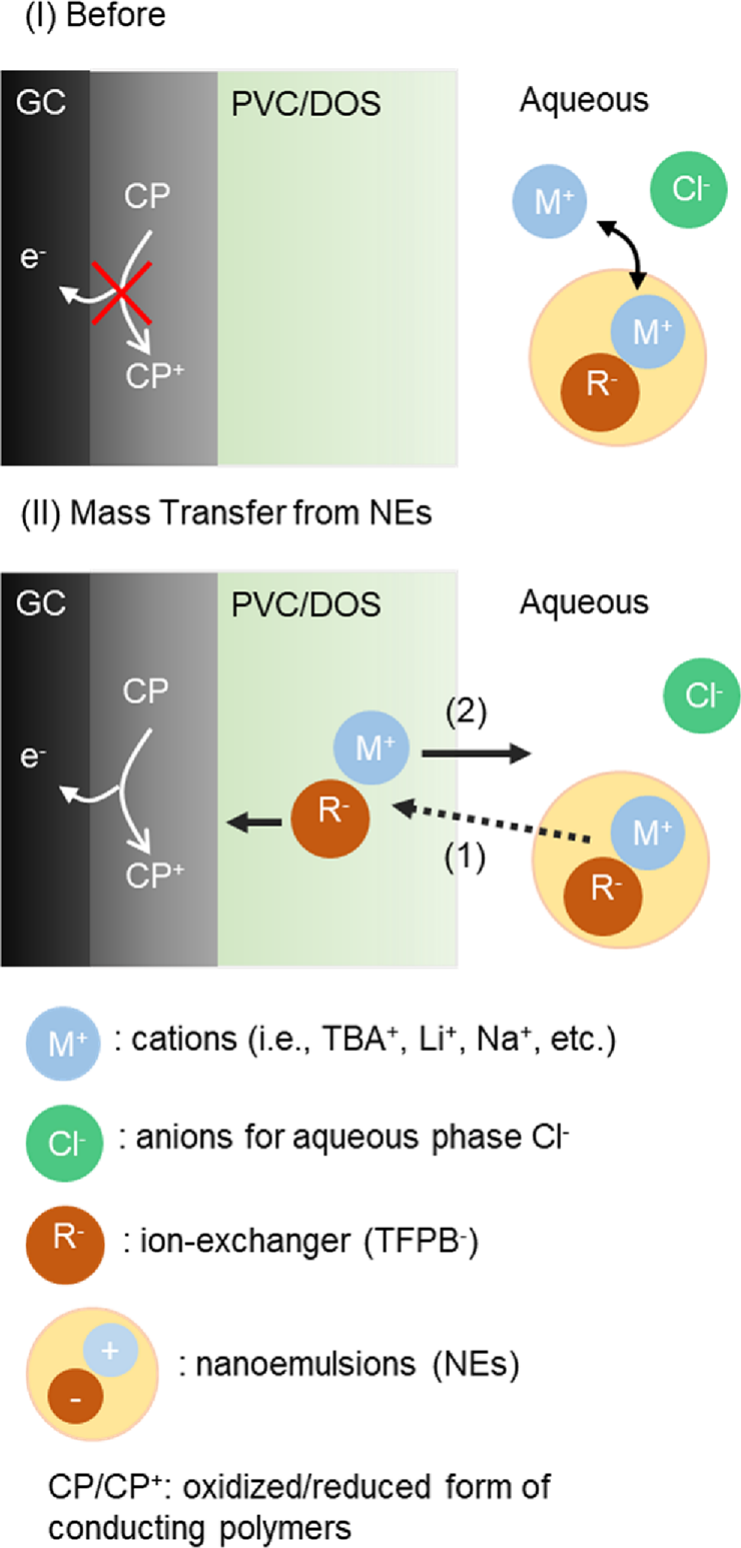
Mechanism of Monitoring the Mass Transfer of a Lipophilic Cation-Exchanger Salt R^-^M^+^ from a Nanoemulsion into a Thin Pristine Membrane where I and II Show the Membrane Composition Before and After the Start of the Mass Transfer Process from Emulsion

Scheme 2 illustrates the principle used here for monitoring the mass transfer from a nanoemulsion phase to the membrane. In the absence of doped lipophilic cation-exchanger no faradaic electrochemistry is observed (Scheme 2 top). During doping, TBA^+^TFPB^–^ transfers from the nanoemulsion into the pristine membrane (step 1 in bottom plot). Once extracted, TFPB^–^ may facilitate the redox reaction of the transducer (PEDOT-C_14_)^21^ by providing its counter ion and allowing TBA^+^ to transfer from the membrane into the aqueous solution (step 2). Consequently, the doping process may be followed by a current increase originating from an exhaustive cation transfer from the membrane.^22^

**Figure 1:**
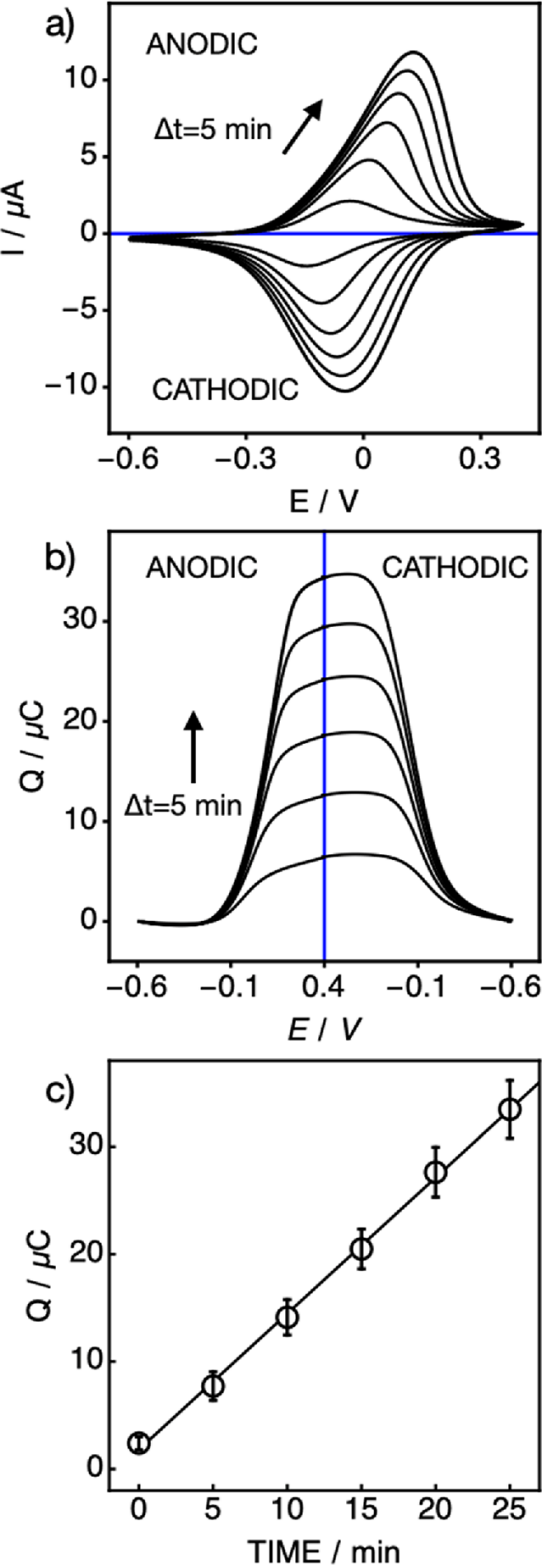
Transfer process at 100 rpm rotating speed with DMF-based NEs encapsulated with TFPB^–^ and monitored by thin-film cyclic voltammetry. (a) Consecutive voltammograms at 5 min intervals during emulsion doping, giving increasing currents with time. (b) Corresponding integrated charge (coulovoltammograms) for the experiment shown in a. (c) Resulting integrated charge as a function of doping time, giving a flux of 3.3 ± 0.2 pmol cm^–2^ s^–1^ of the ion-exchanger salt.

The electrochemical observation of the doping process is shown experimentally in Figure 1 for the transfer of the lipophilic cation-exchanger salt TBA^+^TFPB^–^ (tetrabutylammonium tetrakis[3,5-bis-(trifluoromethyl)phenyl]borate) in DMF-based NEs into a PVC-DOS membrane. The aqueous solution contained 10 mM TBACl, while the electrode was rotated at 100 rpm. The corresponding peak current was found to increase continuously with increasing nanoemulsion doping time. The integration of this current over time gives the accumulated charge. From the known molar mass and Faraday’s law, the corresponding flux into the membrane is then found (Figure 1b). From the slope of Figure 1c, a flux of 3.3 ± 0.2 pmol cm^–2^ s^–1^ is obtained.

In Figure 1, the peak separation increased from 141 to 246 mV over 25 min of nanoemulsion uptake, but the width at half peak current (anodic peak) was maintained at about 293 mV during the entire experiment. This is attributed to the oxidation process of the underlying conducting polymer, involving relatively slow structural changes that result in a small standard rate constant. TBA^+^ was chosen as model reference cation because it does not chemically interact with the PEDOT-C_14_ layer.^23^ Importantly, however, the charges of the anodic and cathodic peaks remained very similar (the differences were ca. 0.3 μC), suggesting that peak separation and broadening has no important bearing on the charge versus time information sought here. The total charge calculated from the anodic/cathodic current grew to 38.3 ± 2.5 μC, accounting for 7.2% of the approximate charge of PEDOT-C_14_ (534 ± 31 μC). Prolonged doping with TFPB^–^ will eventually saturate the resulting charge, which will at that point be limited by the redox capacity of PEDOT-C_14_ (Figure S1).

As shown above, thin-film coulovoltammetry (TFCV) gives information on the doping rate of lipophilic ion-exchangers and ionophores from the nanoemulsion phase. Earlier work assumed that the entire nanoemulsion phase is transferred together with the ion-exchanger salt into the membrane upon contact (mechanism b).^4^ If so, the data in Figure 1 would suggest a total mass transfer rate of 95 ng cm^–2^ s^–1^ (see the Supporting Information for calculations).

**Figure 2:**
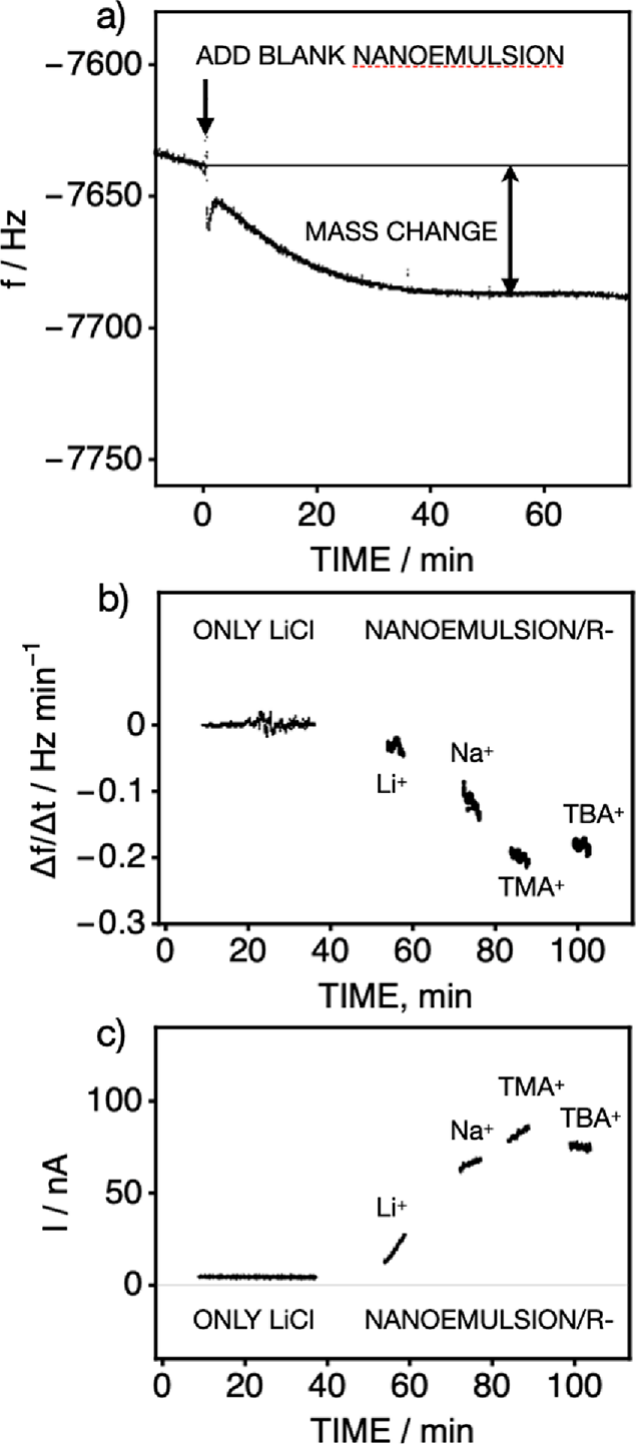
(a) An emulsion, free of TFPB^–^, is added to solution at the indicated time, giving the indicated frequency change that translates into a small mass change of 0.02 ng cm^–2^ s^–1^ indicating minimal fusion between the nanoemulsion phase and the membrane. (b) Frequency change (mass uptake) observed by QCM and (c) the corresponding current with time at 0.4 V upon introducing DMF-based nanoemulsions containing TFPB^–^. The lipophilicity of the salt was successively increased by adding electrolytes of increasing lipophilicity (LiCl, NaCl, TMACl, and TBACl as shown) at a 10 mM concentration to solution. The resulting doping rate does not depend on the lipophilicity, suggesting a mass transport-limited process.

To compare, the mass uptake rate was evaluated experimentally by EQCM, as shown in Figure 2. The PVC-DOS membrane spin-coated on the resonator is sufficiently thin (ca. 200 nm; see ref (24)) to mimic an ideal mass layer. The mass changes on the quartz surface were therefore estimated by the Sauerbrey equation, that is, eq 1 (with *f*_0_: the resonator frequency, 6 MHz; *A*: active electrode area, 0.38 cm^2^; ρ_q_: density of quartz, 2.65 g cm^–3^; and μ_q_: shear modulus of quartz, 2.95 × 10^11^ g cm^–1^ s^–2^):^25^
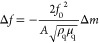
1Nanoemulsions without TFPB^–^ were injected into the EQCM cell after a stabilization time of 150 min (Figure S2) to account for water uptake into the membrane.^26^ From the frequency change in Figure 2a, the transfer rate was calculated as 0.02 ng cm^–2^ s^–1^ (from the red line shown), 3 orders of magnitude lower than the flux calculated if the entire mass of the nanoemulsion were to be transferred (94 ng cm^–2^ s^–1^). As the mass of the membrane barely changed upon nanoemulsion addition, one may conclude that direct fusion was not occurring and mechanism b in Scheme 1 is not applicable. From the same data, loss of the membrane matrix from dissolution may also be excluded. This is different from earlier findings showing partial dissolution of PVC by undiluted DMF, a much harsher condition.^27^ To confirm, the same experiment was also performed without a PVC layer, just with PEDOT-C_14_ directly exposed to a DMF-based nanoemulsion containing TFPB^–^. As shown in Figure S3, no evidence for doping is observed in the absence of the overlaying membrane. This is additional confirmation of the fact that the PVC membrane remains in place during doping.

A comparison was also made with nanoemulsions that were stabilized with the surfactant CHAPS, a zwitterionic surfactant confirmed not to partition into PVC-DOS membranes.^28^ The same general results were obtained; see Figure S2. Furthermore, the particle size remained constant for two days (Table S2), showing that coalescence between particles can be excluded as well.

Since the encapsulated components clearly do not enter the membrane by fusion, one may postulate that the aqueous phase might be involved as the intermediate phase in a two-step partitioning process (Scheme 1c). If so, the rate should be a function of the lipophilicity of the doping compound. A highly lipophilic compound should remain in the nanoemulsion phase because its partitioning into the water phase is completely blocked. As long as the affinity of the compound for the membrane is high relative to water, successively lower lipophilicity should facilitate aqueous partitioning.

To test this hypothesis, the counter ion of TFPB^–^ was successively changed from Li^+^, Na^+^, TMA^+^, and, finally, to TBA^+^. These cations exhibit lipophilicities that differ by orders of magnitude^23^ and result in cation-exchanger salts of dramatically increasing lipophilicity.^29^ One would therefore expect the partitioning of the most lipophilic cation-exchanger salts (with TMA^+^ and TBA^+^) to be highly suppressed relative to the more hydrophilic ones.^18,30^ However, as shown in Figure 2b for the frequency change (top) and corresponding current (bottom), the lipophilicity of the electrolytes does not appreciably alter the transfer rate. After an initial time to reach the steady state with 10 mM LiCl, the rate remained the same, even with the most lipophilic cations.

**Figure 3:**
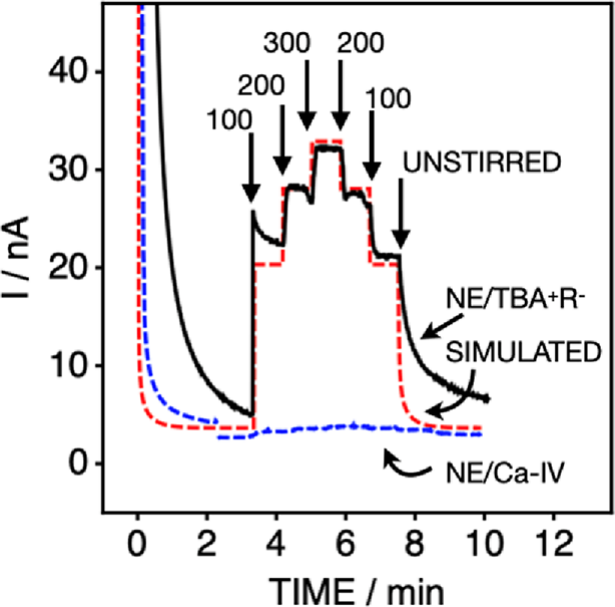
Ion transfer amperometry at a thin membrane for the mass transfer of nanoemulsions containing TBA^+^TFPB^–^ as cargo and a 10 mM TBACl solution as a function of the indicated electrode rotation speed (in rpm) to control the diffusion layer thickness. The solution is quiescent for the initial 3.5 min period. The experimental data (black trace) compares well to theory (red dashed line). In contrast, the flux observed for calcium ionophore uptake from the emulsion phase (Ca-IV, blue dashed line) is much smaller than that predicted based on diffusional mass transport.

As Table S1 shows, the data from EQCM and chronoamperometry all give an approximate average transfer rate of 35 fmol cm^–2^ s^–1^ (31 pg cm^–2^ s^–1^) under otherwise the same experimental conditions. This suggests that the observed uptake rate may be limited by the diffusional transport rate of the nanoemulsion to the membrane surface (Scheme 1a). By defining the diffusion layer thickness using a rotating disk electrode, one may evaluate whether the transfer process is indeed limited by diffusion (Figure 3). The time-dependent flux can be calculated by a numerical simulation (see the Supporting Information)^31^ using the following equation based on an adapted version of Fick’s first law
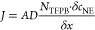
2where *J* is the diffusional flux (mass transfer rate), *A* is the electrode area of 0.071 cm^2^, *D* is the diffusion coefficient of 5 × 10^–8^ cm^2^·s^–1^, *N*_TFPB_ is the number of TFPB molecules in each nanoemulsion particle (4737), and *c*_NE_ is the molar concentration of nanoemulsions in solution (23.8 pmol cm^–3^). Consequently, the concentration of TFPB in solution is 0.1 μmol cm^–3^, which is calculated from *N*_TFPB_ × *c*_NE_. The nanoemulsion diffusion coefficient was estimated by DLS; see Table S2. Based on eq S12, the diffusion layer thicknesses are calculated as 5.9, 4.2, and 3.4 μm for the three rotating speeds (the small values are explained by the low diffusion coefficients of the nanoemulsions). The predicted mass transfer rates are then equal to 2.9, 4.1, and 4.8 pmol cm^–2^ s^–1^ at 100, 200, and 300 rpm rotating speeds, respectively. The simulation is shown as a red trace in Figure 3 with fluxes that agree well with the corresponding experimental data (3.2, 4.2, and 4.8 pmol cm^–2^ s^–1^) for the transfer of the TBA^+^TFPB^–^ salt, shown in black. Figure S4 shows additional experiments by cyclic voltammetry, giving fluxes that range from 2.9 pmol cm^–2^ s^–1^ (100 rpm) to 7.3 pmol cm^–2^ s^–1^ (500 rpm) and are found to be linear with the square root of the electrode rotation speed, as expected from eq S12.

**Figure 4:**
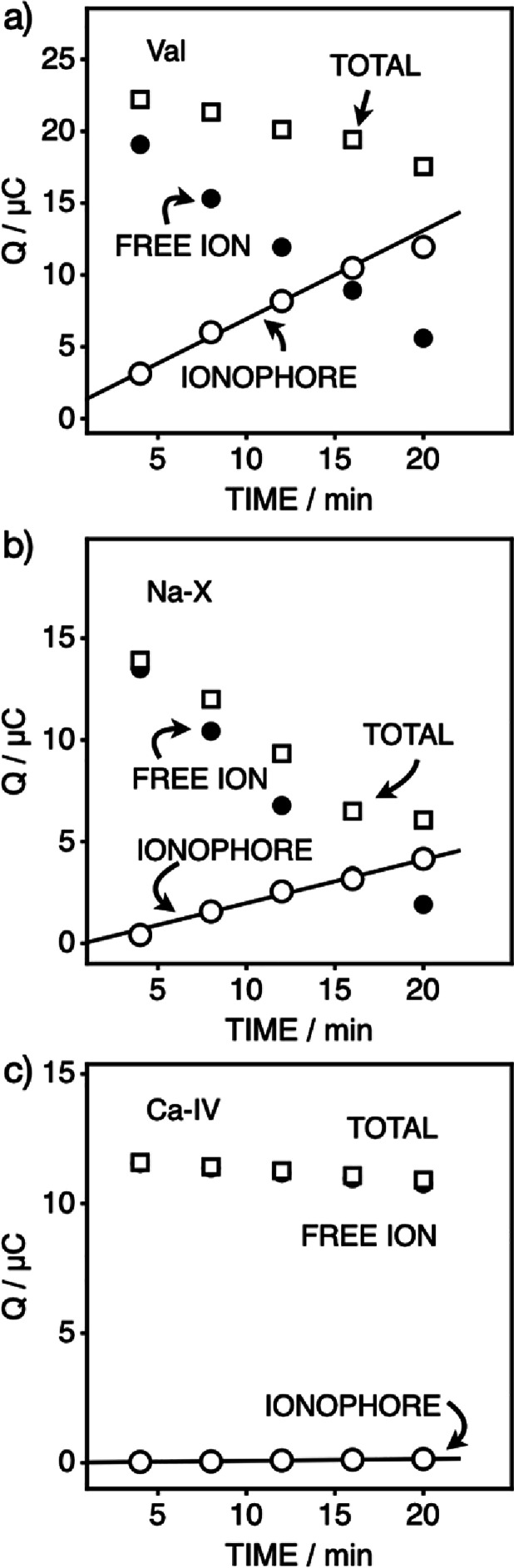
Accumulated charge with time from ion transfer cyclic voltammograms for a thin-layer membrane containing 100 mmol kg^–1^ NaTFPB upon uptake of the ionophores (a) K-I, (b) Na-X, and (c) Ca-IV from the emulsion phase. Shown are the total charge for the two ion transfer peaks (squares), indicative of the cation-exchanger loss from the membrane, and the charge for the uncomplexed (so-called free) ion (black circles), which should decrease as the membrane takes up ionophores. Open circles indicate the charge for the ionophore-bound cation transfer peak, which is found to increase linearly with time and indicates the uptake rate for the ionophore.

The calculated fluxes may now be used to predict the doping rate of components into ion-selective membranes. Previous work demonstrated the doping of a membrane containing 5 mM cation-exchanger with an emulsified lipophilic anion-exchanger.^9^ This resulted in a drastic potential change after a doping time of 0.52 h, indicating the expected change of permselectivity when the anion-exchanger started to be in excess over the cation-exchanger. Assuming a 300 rpm rotating speed, this time is estimated as 0.54 h based on the available data in this study (Figure 4b), which is indeed similar. Note, however, that the experimental conditions between the two studies were sufficiently different that a quantitative comparison should not be made.

While the uptake rate of highly lipophilic cation-exchanger salts is clearly limited by diffusional mass transport, one may expect species of even higher lipophilicity to eventually behave differently. For this, the transfer of three ionophores of different lipophilicities was evaluated. The potassium carrier valinomycin (K-I) has a calculated logarithmic octanol–water partition coefficient of log *P* = 9.5; see Table 1. The sodium ionophore 4-*tert*-butylcalix[4]arene-tetraacetic acid tetraethyl ester (Na-X, log *P* = 16.3) and the calcium ionophore *N*,*N*-dicyclohexyl-*N*′,*N*′-dioctadecyl-diglycolic diamide (Ca-IV, log *P* = 21.0) exhibit higher lipophilicities.

**Table 1:** Calculated Logarithmic Octanol–Water Partition Coefficients (log *P*) and Experimental Logarithmic Mass Transfer Rates (log *J*) Measured by TFCV of the Indicated Compounds at a 100 rpm Rotating Speed

component	K-I	TBA^+^TFPB^–^	Na-X	Ca-IV
log *P*	9.5	14.6	16.3	21.0
log *J*(mol cm^–2^ s^–1^)	–11.8 ± 0.3	–11.5 ± 0.0_3_	–12.4 ± 0.2	–13.7 ± 0.3

Thin-film coulovoltammetry (TFCV) can also be used to visualize the uptake of neutral ionophores as shown in Figure 4. Based on earlier research,^4,7,32−34^ ion–ionophore interactions give rise to a peak shift for the outward cation transfer inhibited by the ionophore relative to the uncomplexed ion, requiring a more positive potential. The relative charge from these two peaks can be used to monitor ionophore uptake if the cation-exchanger in the PVC membrane is kept at a sufficiently high concentration (here at 100 mmol kg^–1^).

From the data shown in Figure 4a for the uptake of valinomycin, a linear increase of charge with time of 0.62 μC min^–1^ (giving a flux of 1.5 pmol cm^–2^ s^–1^; Table 1) was observed (Figure S5a,b). For the sodium ionophore of higher lipophilicity, the data in Figure 4b gave a threefold smaller flux (0.21 μC min^–1^ or 0.5 pmol cm^–2^ s^–1^; Figure S5c,d and Table 1). In contrast, the much more lipophilic calcium ionophore gave a two-orders of magnitude smaller doping rate as shown in Figure 4c (0.007 μC min^–1^ and 17 fmol cm^–2^ s^–1^; Figure S5e,f and Table 1). It is evident that the transfer of species of extremely high lipophilicity is kinetically hindered and no longer limited by diffusional mass transfer. For the calcium ionophore, this was directly confirmed by performing rotating disk electrode experiments; see the blue trace in Figure 3. The experimental chronoamperometric data are much smaller than theoretically expected for a diffusion limitation in contrast to the transfer of cation-exchanger salts. It may be concluded that species exhibiting an octanol–water partition coefficient higher than approximately 15 orders of magnitude exhibit a transfer rate from the emulsion phase that is limited by the transfer through the aqueous nanogap between the emulsion and membrane phases.

One may note that there is some evidence in Figure 4a,b for the transfer of the cation-exchanger salt in the opposite direction, from the membrane to nanoemulsion. The leakage rate for the ion-exchanger from the membrane may be followed by the total charge (shown as squares) for the transfer of so-called free and complexed cations from the membrane over time. Xie et al. showed evidence of a kinetically hindered transfer of rhodamine dye between three phases, which may be similar to the situation described here.^35^ Surprisingly, the calcium ionophore appears to help limit the outward TFPB^–^ transfer rate. One may argue that this is related to the large formation constant of the calcium-ionophore complex, which increases the cation-exchanger lipophilicity by reducing the concentration of the uncomplexed counter ion of the cation-exchanger.^29^ However, as shown in Figure S6, the transfer rates from the emulsion phase containing the cation-exchanger and calcium ionophore into the membrane are similar to those in the case where TFPB^–^ is initially present in the membrane. For systems where diffusional mass transfer is not rate-limiting, it therefore appears to be difficult to predict the transfer rate based on structural or binding information.

## Conclusions

Drawing inspiration from previous work on ion-selective membranes doped from the emulsion phase, we developed a thin-film coulovoltammetry (TFCV) method to allow for the quantitative monitoring of the mass transfer from DMF-based nanoemulsions loaded with a range of sensing components as cargo. The transfer was performed into a thin membrane on a solid-state electrode modified with PEDOT-C_14_ as an ion-to-electron transducer. With complementary EQCM data, the mass transfer mechanism was shown not to involve complete fusion of the nanoemulsion with the membrane. The peak positions on the cyclic voltammogram helped us to distinguish the transfer of the ion-exchanger from that of ionophores. This was used to study the uptake rate of a wide range of components, including cation-exchanger salts with counter ions of varying lipophilicities and three ionophores of widely different lipophilicities. For the TFPB salts and the potassium ionophore valinomycin, the transfer rates are dictated by diffusional mass transport of the emulsion phase. For these cases, it may be possible to predict the doping rates of ion-selective membranes by known experimental parameters. Highly lipophilic components with octanol–water partition coefficients higher than 15 orders of magnitude are no longer subject to diffusional mass transport limitation, suggesting that the aqueous phase strongly attenuates transfer kinetics.

## Supporting Information Available

Detailed experimental information, calculation of the nanoemulsion concentration, analysis of mass transfer rates, and diffusion simulation (PDF)
