# Supplementary material for: Mass Transfer from Ion-Sensing Component-Loaded Nanoemulsions into Ion-Selective Membranes: An Electrochemical Quartz Crystal Microbalance and Thin-Film Coulometry Study
Source: ACS Meas Sci Au. 2022 Oct 4;3(1):45–52. doi: 10.1021/acsmeasuresciau.2c00053 (PMC9936608; doi:10.1021/acsmeasuresciau.2c00053)
Supplement: Supplementary file 1 — tg2c00053_si_001.pdf [file tg2c00053_si_001.pdf]

Supporting Information for:

Mass Transfer from Ion Sensing Component-Loaded Nanoemulsions into Ion-Selective Membranes: An Electrochemical Quartz Crystal Microbalance and Thin Film Coulometry Study

Canwei Mao, Yoshiki Soda, Kye J. Robinson, Tara Forrest, Eric Bakker*

Department of Inorganic and Analytical Chemistry, University of Geneva, Quai Ernest-Ansermet 30, CH-1211 Geneva, Switzerland

*Email: eric.bakker@unige.ch

1. 1. Formulas and Constants

Avogadro's constant N_A: 6.02×10^{23}
Number of moles of substance: n_m
The number of particles N is defined as:

$N = n_m N_A$ S1

Where N is the number of nanoemulsion (NE) particles in solution. To obtain N in EQCM experiment, we first calculated the size V_{NE} of the NE particles,

$V_{NE} = (4/3)\pi r^3$ (radius of NEs is obtained from Table S2). S2

With the known volume of solvent (DMF) V_S, the number of NEs particles in solution is

$N = \frac{V_S}{V_{NE}}$, S3

We may obtain the concentration of NE particles in solution with eq. S1,

$c_{NE} = \frac{n_m}{V_S}$ S4

In the same manner, the N_{TFPB} of TFPB in each NE particle will be

$N_{TFPB} = \frac{m_{TFPB}}{M_{TFPB} N_A N}$ S5

where m_{TFPB} and M_{TFPB} are the mass and molar mass of TFPB.

The transfer rate (flux) of TFPB can be described on the basis of Fick's law,

$$J_i = -D\frac{c_i}{\delta} \quad \text{S6}$$

where J_i represents the flux of species i and c_i is its concentration. We can now calculate the flux of TFPB encapsulated in NEs by eqs S4-6 as:

$$J_{TFPB} = -\frac{DN_{TFPB}c_{NE}}{\delta} \quad \text{S7}$$

where δ is the diffusion layer thickness.

1. 2. Simulations

According to Fick's law, we can describe the current from the ion flux by the discretized concentration profile of the diffusion, described by eq. S8 and S9:

$$I = -N_{TFPB}AFJ(x,t) = -AFD\frac{N_{TFPB}\cdot\Delta c_{NE}}{\Delta x} \quad \text{S8}$$

$$c_{n,t+1} = c_{n,t} + \tau\left(c_{n-1,t} - 2\,c_{n,t} + c_{n+1,t}\right) \quad \text{S9}$$

where charge number of TFPB$^-$ z is -1, A denotes the area of electrode surface. k as the iteration of time defines the resolution of the diffusion profile, τ is the model diffusion coefficient set as 0.45 and $c_{n,t}$ represents the concentration of TFPB$^-$ in the NEs (calculated by SI-1.1) at the position number n of the increment Δx away from the electrode surface at time t). We can then describe the concentration profile for the diffusion process. The parameters of the model simulation from eq. S9 and S10 are then inserted into eq S8 and the resulting current is predicted from eq S11.

$$\tau = \frac{Dk\Delta t}{\Delta x^2} \quad \text{S10}$$

$$I = -\frac{FAD(c_{0,t}-c_{1,t})}{\Delta x} = -\frac{FA(-c_{1,t})\sqrt{DkD_m}}{\sqrt{\Delta t}} \quad \text{S11}$$

For the boundary condition in the numerical simulation, the actual thickness of the spin-coated membrane (sub-nanometer) is similar to the model variable Δx (0.1 µm when k=0.001). Thus, diffusion is neglected in the membrane phase. The concentration of the component in the bulk emulsion solution is defined in the Experimental Section. In the membrane, it is considered zero during the equilibrium state because the transducer (PEDOT-C_{14}) is here oxidized and positively charged (potential applied constantly at 0.4 V) and accumulates the component (TFPB$^-$) transferred from the emulsion solution unless it exceeds the total charge of PEDOT-C_{14}. After this equilibrium state, the system will break down as shown in Figure S1 owing to the uncertainty of dynamic component transfer.

The diffusion layer thickness δ was controlled by the rotating speed ω (ranging from 100 to **5**00 rpm) and calculated by eq. S12 (η kinematic viscosity: 1.004 cm^2/s):

$$\delta = 1.61\,D^{1/3}\omega^{-1/2}\eta^{1/6} \quad \text{S12}$$

Subsequently, one can calculate the maximum position number at each different diffusion layer thickness adapted to the model by $n_{max} = \delta/\Delta x$, and the smaller Δx one defines, the closer the value will approach reality (Figure S7). Note that changes in the ohmic drop influences the baseline in cyclic voltammetry, because the PVC-DOS membrane is initially blank and contains no doped lipophilic electrolyte, giving rise to a high resistance that is gradually reduced by doping with cation-exchanger salt from the emulsion phase**.**

TFCV method verifies the simulation model as well by giving a linear relationship between the square root of the rotating speed and the observed transfer rate (from eq S12, see Figure S4). This relationship (Figure S4) can facilitate the estimation of the transfer rate for a similar membrane doping system. The diameter (d) of NEs alters the diffusion coefficient as predicted by the Stokes-Einstein equation ($D = kT/3\pi\xi d$, k: the Boltzmann constant, T: the absolute temperature, ξ:dynamic viscosity). This was adapted in eq. S8 and S9, so that a linear function between transfer rate and its diameter may be taken into account.

2. Additional Results

Table S1. Observed overall mass transfer rate by QCM and transfer rates of just the active ion-exchanger component observed by chronoamperometry at a thin membrane for the indicated nanoemulsion (NE) composition.

DMF-based NEs	QCM		Chronoamperometry		Thin-film coulovoltammetry	
	Δf mHz/min	Transfer Rate pg/(cm^2·s)	ΔQ nC/min	Transfer Rate pg/(cm^2·s)	ΔQ nC/min	Transfer Rate ng/(cm^2·s)
Li	1.2	-0.3 ± 1.0	4.4	0.0_1 ± 0.0		
Li+NEs	-33.2	6.8 ± 1.3	19.2	7.4 ± 1.4		
Na+NEs	-115.9	**23.7 ± 2.2**	66.0	**25.6 ± 0.4**		
TMA+NEs	-198.7	**40.6 ± 1.1**	82.2	**31.8 ± 0.7**		
TBA+NEs	-181.0	**37.0 ± 1.1**	75.2	**29.1 ± 0.2**	755.5	1.6 ± 0.5

PS. The bold face give the average transfer rate **31.31 pg/(cm^2·s)**, which is lower than the ideal transfer rate of ca. 1 ng/(cm^2·s) as δ = 50 μm (at 0.3 rpm). It might be caused by the O-ring attached with the membrane of EQCM cell absorbing the DMF solvent of NEs.

Table S2. Nanoemulsion size measured by Dynamic Light Scattering (DLS).

DLS	Exp1		Exp2		Exp3		Overnight	Over 2 days
	Before	After	Before	After	Before	After		
z-average (nm)	174±31	115±0.80	99±2	107±5	100±7	107±13	112±11	0
diffusion coefficient (μm^2/s)	3.6±0.30	4.6±0.03	5.4±0.01	4.9±0.03	5.28±0.03	4.8±0.05	4.7±0.01	0.004±0.003

Note: 1) the particles are measured before and after the experiment, 2) content in red indicates the particles are instable and fell apart.

Table S3. Resolution variations for the diffusion model variables n=δ/Δx with 100, 200 and 300 rpm corresponding to different diffusion layer thicknesses. In bold is the condition for the model shown in the main text.

Rotating Speed / rpm		100	200	300
Actual diffusion layer thickness δ / μm		2.76	1.95	1.59
n = δ / Δx	k = 1	1 (0.83)	1 (0.58)	0 (0.48)
	k = 0.1	3 (2.61)	2 (1.85)	2 (1.51)
	k = 0.01	8 (8.27)	6 (5.84)	5 (4.77)
	**k = 0.001**	**26 (26.14)**	**18 (18.48)**	**15 (15.09)**

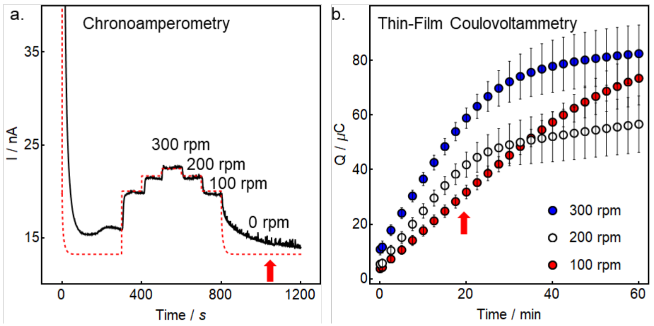

Figure S1. a) Ion transfer chronoamperometry at a thin membrane as a function of electrode rotation rate, in contact with a nanoemulsion containing TFPB. In red: simulated behavior. b) Thin membrane film coulovoltammetry as a function of time upon NE contact, showing how the integrated charge increases with time. After ca. 20 min, the TFPB transferred from the nanoemulsion starts to approach the redox capacity of the conducting polymer, resulting in a deviation from linearity.

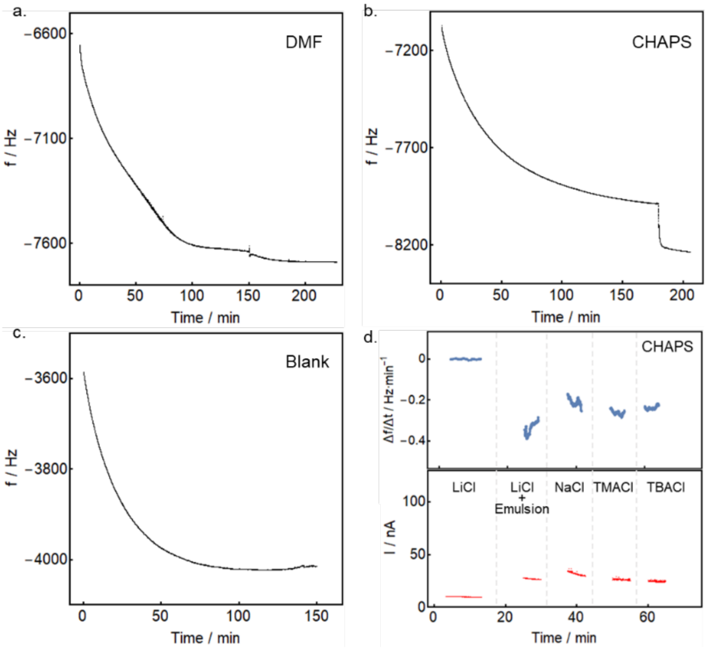

Figure S2. Conditioning the membrane for water uptake in a) DMF-, b) CHAPS-based NEs system and c) solution without NEs; d) EQCM experimental results of mass transfer for CHAPS-based NEs in solution with different lipophilic electrolytes.

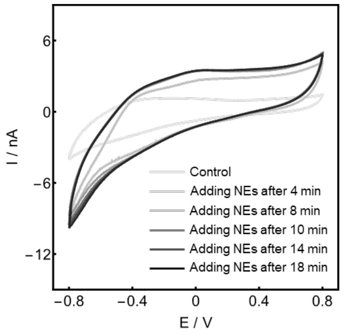

Figure S3. Time evolution of CVs after adding DMF-based nanoemulsions (NEs) containing $TFPB^-$ to a solution with 10 mM TBA^+ with an electrode electrodeposited with PEDOT-C_{14}, without any PVC-DOS membrane.

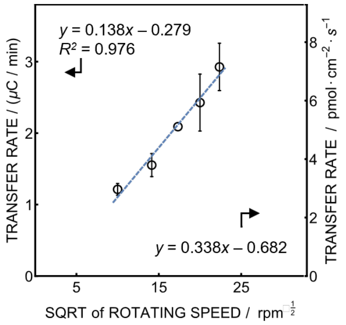

Figure S4. Mass transfer rate measured by thin-film coulovoltammetry method for different rotating speeds. Linearity indicates diffusional mas transport limitation.

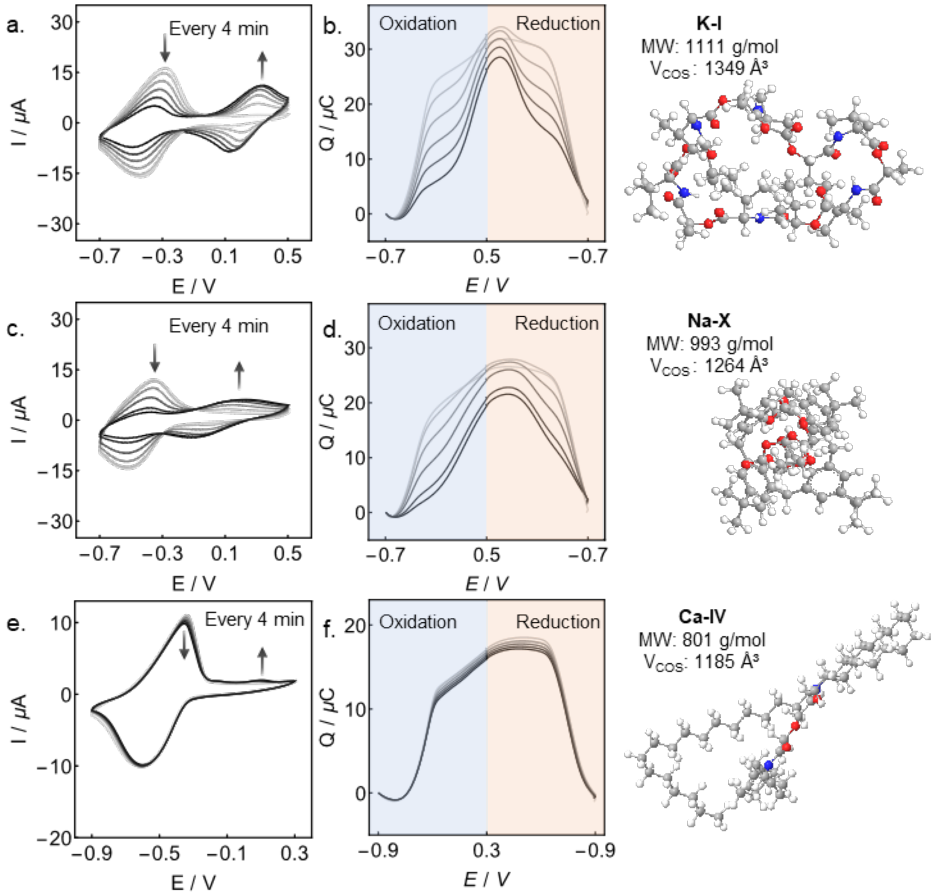

Figure S5. Left panels: cyclic voltammograms of PVC-DOS membrane initially containing cation-exchanger only, doped with NEs encapsulated with the indicated ionophores, a) K-I, c) Na-X and e) Ca-IV. Middle: corresponding accumulated charge. Left: 3D structure of ionophore with indicated molar mass and molecular volume.

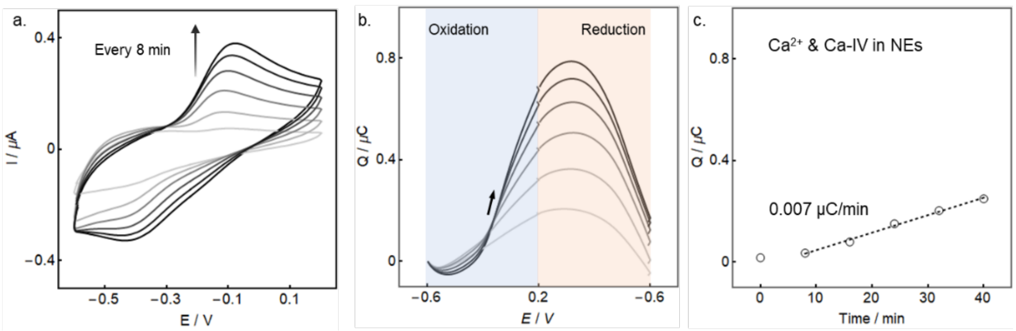

Figure S6. a) Cyclic voltammograms of PVC/DOS membrane initially containing no cation-exchanger or ionophore, taking up the ionophore Ca-IV and TFPB- from NEs, recorded every 8 min, together with b) the corresponding coulovoltammograms. Line shading from light grey to black indicates increase in time). c) Accumulated charge (average charge for the anodic and the cathodic currents) corresponding to complexed ion transfer at the indicated time after contact with NEs encapsulating Ca-IV and $TFPB^{-}$.

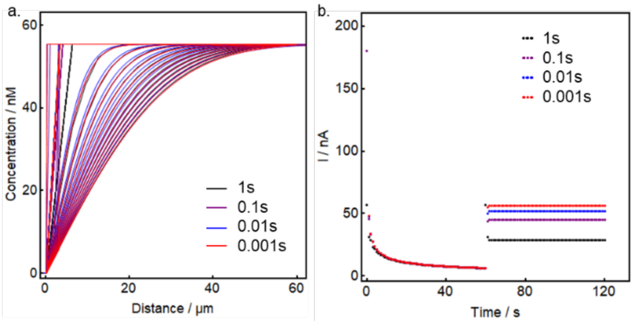

Figure S7. Calculated variations of resolution in the numerical simulation of mass transport, based on time iteration changes from 0.001 s to 1 s. The diffusion layer thickness is diminished by 5 µm after 60 s, shown a) as a profile of concentration vs. distance from the electrode surface and b) in the corresponding calculated current response vs. time.
